# Integrating Quality of Life in the Care Pathway of Cancer Patients Undergoing Immunotherapy Treatment: Descriptive, Cross-sectional Survey of an Online Patient Community's Experiences and Expectations

**DOI:** 10.2196/25792

**Published:** 2022-01-11

**Authors:** Ophélie Wilczynski, Anthony Boisbouvier, Lise Radoszycki, François-Emery Cotté, Anne-Françoise Gaudin, Hervé Lemasson

**Affiliations:** 1 Carenity Paris France; 2 Bristol-Myers Squibb France Rueil-Malmaison France

**Keywords:** cancer, quality of life, immunotherapy, patient community, patient satisfaction

## Abstract

**Background:**

New cancer treatments, such as immune checkpoint inhibitors (ICIs), can improve survival and health-related quality of life (HRQoL) in patients with cancer. Although long-term monitoring of HRQoL has been shown to improve survival, integration of HRQoL into everyday practice remains poorly documented.

**Objective:**

This study describes experiences and expectations of patients treated with ICIs regarding a discussion of HRQoL with health care professionals (HCPs) in cancer management.

**Methods:**

This cross-sectional study was conducted in an online patient community (Carenity) in France. Patients treated with ICIs for cancer, included between September 2018 and January 2019, completed a questionnaire to assess the involvement of HCP in a discussion of HRQoL and when and what was discussed.

**Results:**

Of 82 patients included (mean age: 56.9 years, 95% CI 54.2-59.6; 46 [56%] male; 34 [41%] with lung cancer), 62 (76%) reported discussing HRQoL at least once with HCPs, mainly general practitioners (54/82, 66%), oncologists (53/82, 65%), and hospital nurses (50/82, 61%). Around half (45/82, 55%) of the patients were satisfied with these discussions. Discussions with the oncologist were at the patient’s initiative (34/53, 64%). Discussions occurred primarily during follow-up visits (40/62, 65%), when adverse events occurred (30/62, 48%), and at treatment initiation (27/62, 32%). The most discussed dimensions were symptoms (48/62, 77%) and physical well-being (43/62, 69%). With respect to expectations, 54/82 (66%) patients considered oncologists as the most important HCPs for discussing HRQoL. These discussions were desirable throughout the care pathway, particularly at diagnosis (63/82, 77%) and when treatment was initiated (75/82, 92%) or changed (68/82, 83%). All HRQoL dimensions were considered important to discuss.

**Conclusions:**

With only around half of the patients satisfied with HRQoL discussions, impactful HRQoL integration in clinical practice is critical. According to patients, this integration should involve mainly oncologists and general practitioners, should happen at every step of the care pathway, and should be extended to dimensions that are currently rarely addressed.

## Introduction

Health-related quality of life (HRQoL) is a critical feature of the life of patients with cancer, and a number of instruments have been developed for evaluating this over the past 40 years. These include general HRQoL instruments that are not specific to cancer but can be used to compare HRQoL between cancer and other diseases, as well as instruments that are specific to cancer [1]. Such cancer-specific instruments include the European Organisation of Research and Treatment of Cancer Quality of Life Questionnaire (EORTC-QLQ) family of questionnaires [2] and the Functional Assessment of Cancer Therapy (FACT) family [3], of which individual versions have been designed for specific types of cancer. These disease-specific HRQoL measures are used systematically as outcome measures in clinical trials but may also be used to support discussions of HRQoL in the everyday care of patients with cancer.

In cancer, HRQoL is impacted by disease symptoms as well as by side effects and constraints associated with therapy. Moreover, HRQoL can change rapidly and sometimes unpredictably over the course of the disease. Preserving the HRQoL of patients with cancer is a major goal of clinicians and health authorities [4-6]. For this reason, building and maintaining an open dialogue between patients and health care professionals (HCPs) is essential in order to evaluate the patient’s HRQoL adequately and to promptly address any issues that may arise. Systematic monitoring of the patient’s perceptions of HRQoL has also been shown to be of benefit in terms of symptom management [7], satisfaction with their care [7], a greater use of supportive care [7], improvement in clinician-patient communication [8,9], and improved overall survival [10-13], since it allows, among other potential advantages, timely adaptation of treatment in the case of symptom progression or emergence of treatment side effects.

The introduction of immune checkpoint inhibitors (ICIs) over the past decade has represented a major advance in the treatment of many types of cancer, allowing sustained recovery and, for some tumors, potentially elimination of disease in a significant proportion of patients [14,15]. By providing patients with a survival benefit [16] and a better tolerance profile compared to traditional chemotherapy [17], treatment with ICIs has become an attractive therapeutic alternative for many types of cancer. In terms of HRQoL, the experience of cancer patients treated with immunotherapy may differ from that of patients receiving standard chemotherapy. Treatment with ICIs may be associated with a different profile of response compared with standard chemotherapy, due to longer periods of disease stability and the lower incidence of side effects that have an impact on the quality of life (QoL) [17]. Several studies have investigated HRQoL in patients treated with ICIs [18-20] and have shown maintenance of HRQoL over long periods, and even improvement in HRQoL compared to standard chemotherapy in certain patients [18,19,21,22].

Most QoL research with ICIs has been conducted in the context of clinical trials, although some observational studies on long-term survivors who are clinically stable have been reported [23,24]. Potentially deleterious effects of ICI-specific adverse events on HRQoL [25,26] and potentially beneficial effects on social functioning and role integration [27-29] are aspects that would deserve attention. In addition, development of a specific HRQoL measure for cancer patients using ICIs with stable disease could be useful [30]. From an operational and a care perspective, the changes in the treatment paradigm associated with the introduction of ICIs indicate the utility of monitoring HRQoL over the long term in everyday practice. This could provide benefits in optimizing functional outcomes in a timely manner, as well as in contributing to treatment decisions. However, little information is available on how HRQoL is considered by physicians treating cancer patients with ICIs in routine clinical practice.

The objectives of this study were to describe experiences of patients treated with ICIs and their expectations with respect to how the importance of HRQoL in cancer management is considered by HCPs. This includes the description of practices of HCPs in appraising HRQoL with patients currently or previously treated with ICIs, the evaluation of patient satisfaction with their dialogue about HRQoL with their HCPs, and the identification of patient expectations with respect to discussing HRQoL.

## Methods

This study was a descriptive, cross-sectional web-based survey of cancer patients (or their relatives) treated with ICIs who were members of the Carenity cancer community and resident in France. Participation was voluntary. Participants were recruited over 4 months from September 10, 2018, to January 7, 2019.

### Study Population

The study population included participants from the Carenity cancer community. Carenity is an online patient community for people with chronic conditions [31,32]. Patients and caregivers can share their experiences in more than 1200 disease-specific communities, exchange information on the disease and request advice and information. They can also participate in online surveys concerning various aspects of disease perceptions on a voluntary basis and after giving explicit consent. Currently, the cancer patient community on Carenity in France has around 9547 members, of whom 5871 (61.5%) are patients. All Carenity cancer community members were invited to participate in this study.

Participants could either be patients themselves or a relative (or friend) who was prepared to complete the study questionnaire on their own or with the patient. Relatives were asked to complete the questionnaire from the patient’s point of view. In the rest of the manuscript, the data presented represent the characteristics and opinions of the patients, regardless of whether it was the patients themselves or a relative who completed the questionnaire. Participants were eligible for the study if they or their relative were currently or previously treated with an ICI (atezolizumab, durvalumab, nivolumab, pembrolizumab, or ipilimumab).

As members of the Carenity platform, patients or relatives participating in the study provided explicit informed consent to the collection, handling, and keeping of their personal and health data. They were also provided with specific information about the goals and procedures of the study, as well as about the notion of HRQoL, and asked to agree to participate before receiving the study questionnaire. Participants received no incentives to participate in the study, and participation had no impact on their future involvement as Carenity platform members.

### Study Questionnaire

The questionnaire was developed specifically for this study. The HRQoL domains explored are based on constructs in 2 existing validated cancer-specific HRQoL questionnaires (QLQ-C30 [33] and FACT-G [3]). The questionnaire was subsequently tested for clarity and relevance by 2 representatives of the Carenity cancer community.

The study questionnaire started with a set of screening questions to identify the participant as a patient or as a relative and to ensure that the patient had a diagnosis of cancer and was being treated (or had been treated previously) with an ICI. If this was not the case, the participant left the study at this point. Otherwise, they proceeded to the core questionnaire, which took, on average, around 15 minutes to complete.

The core questionnaire consisted of 29 questions for all participants, as well as 3 additional ones to be completed only by relatives answering on behalf of a patient, which were divided into 3 sets, relating to general information, experiences with discussion of HRQoL, and expectations for discussing HRQoL with HCPs. The themes and attributes evaluated during the study are listed by theme in Table 1. The first set of 14 general questions collected data on patient demographics, cancer history, recent treatment (12 months), HCPs consulted, and treatment location. The second set of questions started with an open-ended question asking patients to sum up in 3 words or phrases the aspects of their HRQoL that were most impacted by cancer and its treatments. Participants were then asked whether they had ever discussed QoL with an HCP and when. The period of time covered was not restricted to the period of treatment by ICIs but related to the entire period since the diagnosis of cancer was given. Only patients for whom this was the case completed the other questions in this set. in total, 10 questions collected information about the dialogue between the patient and the medical care team, covering the type of HCP involved, when HRQoL was discussed, the aspects of HRQoL discussed, and satisfaction with the discussions. Finally, all participants, whether or not they had discussed HRQoL, completed the last set of 6 questions about expectations for a dialogue with an HCP about QoL, which covered an identical set of concepts as those explored in the previous set of questions on experiences.

**Table 1:** Information collected during the study.

Attribute studied		Question	Response modality/data analysis	Data presentation
**Perceptions of the impact of cancer on QoL^a^**				
	Impact of cancer on QoL	Can you cite 3 words or expressions that you think best express the aspects of QoL that are impacted by your cancer?	Open question Replies grouped by theme	Number and % of patients citing each theme
**Discussion of QoL with HCPs^b^**				
	Importance of discussing QoL	Do you think that discussing QoL with HCPs is . . . (list)?	Checklist of 5 levels of importance Single response only	Number and % of patients citing each importance level
	Experience of discussing QoL	On what occasion(s) did you discuss QoL with the HCP who looks after you?	Checklist including “Never” Multiple responses possible	Number and % of patients citing each occasion Number of different HCPs identified
	Desire to discuss QoL	You replied that you have never discussed QoL with an HCP. Would you have liked an opportunity to do so?	Yes/No/Don’t know	Number and % of patients replying yes
	Satisfaction with discussions of QoL	Were you satisfied with the way that QoL has been brought up by different HCPs?	Checklist Single response only	Number and % of patients citing each response
	Reasons for satisfaction or dissatisfaction	What was the reason that you were satisfied or dissatisfied?	Open question Replies grouped by theme	Number and % of patients citing each theme
	Opportunity to express yourself	Do you feel that you were able to express yourself about the impact of cancer or cancer treatments on your QoL?	Checklist of 5 response modalities Single response only	Number and % of patients citing each response
**HCPs involved in HRQoL^c^ discussions**				
	Types of HCP discussing QoL	When you consult 1 of the following types of HCP, do you discuss QoL with them?	Checklist of different HCPs with 5 response modalities for each Including “Never/ I don’t consult this HCP” Single response only	Number and % of patients responding often, occasionally, or rarely for each HCP specialty
	Who initiates the discussion?	When you discuss QoL with your oncologist or radiotherapist, who usually initiates the conversation?	Checklist of 5 response modalities Single response only	Number and % of patients citing each response
	Importance of different HCPs	Which HCPs do you think are the most important for talking about QoL?	Checklist of different HCPs Multiple responses possible	Number and % of patients citing each type of HCP
	Other contexts where QoL is discussed	Have you ever discussed your QoL in another context (discussion group, therapeutic education program, etc)?	Checklist of 5 contexts Single response only	Number and % of patients citing each context
**Opportunities for discussing Qo**L				
	Occasions when QoL had been discussed	On what occasion(s) did you discuss QoL with the HCP who looks after you?	Checklist Multiple responses possible	Number and % of patients citing each occasion
	Relative importance of different occasions for discussing QoL	Which occasions do you think are particularly important for discussing QoL with HCPs?	Checklist of different HCPs with 5 response modalities for each Single response only	Number and % of patients citing each occasion
**Dimensions of QoL discussed**				
	Subjects discussed	When you discuss QoL, what are the subjects that you usually discuss?	Checklist Multiple responses possible	Number and % of patients citing each subject Number of different subjects identified
	Relative importance of discussing different subjects	How much importance do you attach to discussing the following subjects with an HCP?	10 cm visual analog scale for each of the 9 subjects	Mean score with standard deviation
**Measures for improving discussions of QoL**				
	Ways to improve paying attention to QoL	How could the medical team involved in your care pay more attention to your QoL?	Open question Replies grouped by theme	Number of citations for each theme
	Specific measures	In your opinion, which are the 3 measures that would be most useful to improve discussions of your QoL?	Checklist of 11 measures 3 responses possible	Number and % of patients citing each measure

^a^QoL: quality of life.

^b^HCP: health care professional.

^c^HRQoL: health-related quality of life.

All questions in this web-based survey were mandatory to access the next question, except for 4 open ones. Skip patterns were used, when appropriate. Most of the questions were single or multiple choice, to which participants responded by ticking boxes. Two questions were in the form of Likert scales, and another asked patients to rate the importance of 9 HRQoL dimensions on a visual analog scale.

### Data Analysis

The data analysis was purely descriptive as no prespecified hypotheses were tested. Responses to multiple-choice questions and Likert scales are presented as frequency counts and percentages with their 95% CIs.

### Ethics

The study was conducted in accordance with good epidemiological practice. As the aim of the study was to determine patient satisfaction, the survey is considered a patient satisfaction survey and does not fall within the scope of French legislation on medical research. For this reason, submission to an ethical committee was not required.

## Results

### Patient Population

A total of 82 questionnaires were fully completed, of which 56 (68%) were completed by the patients and 26 (32%) by a friend or relative. In the latter case, 16 (61%) questionnaires were completed in the presence of the patient. The characteristics of the patients are presented in Table 2. Overall, 46 of 82 (56%) patients were men, and the most frequent cancer types were lung cancer, lymphoma, and skin cancer, which accounted between them for 58 (71%) cases. The remaining cancer types accounted for ≤5 (6%) patients each. The mean age was 56.9 years (95% CI 54.2-59.6), and this was similar across the principal cancer types (58 years for lung cancer and lymphoma and 52 years for skin cancer). The diagnosis of cancer had been made within the previous 5 years for two-thirds of patients. Overall, 62 of 82 (76%) patients had discussed their HRQoL with an HCP, and only these patients completed the set of questions about their experience. Information about 1 patient who had died was provided by a relative.

**Table 2:** Characteristics of study patients (N=82).

Characteristic		n (%)
**Age (years)**		
	18-30	2 (2%)
	31-40	7 (9%)
	41-50	12 (15%)
	51-60	29 (35%)
	61-70	22 (27%)
	>70	10 (12%)
**Gender**		
	Men	46 (56%)
	Women	36 (44%)
**Primary cancer localization**		
	Lung	34 (41%)
	Lymphoma	12 (15%)
	Skin	12 (15%)
	Kidney	5 (6%)
	Prostate	3 (4%)
	Ovarian	3 (4%)
	Leukemia	3 (4%)
	Other^a^	10 (12%)
**Time since cancer diagnosis**		
	0-5 years	65 (79%)
	6-10 years	10 (12%)
	>10 years	6 (7%)
	Do not know	1 (1%)
**Place of treatment in previous 12 months^b^**		
	University hospital	28 (34%)
	Local hospital	27 (33%)
	Private clinic	25 (30%)
	Specialist cancer center	14 (17%)
	Community medical center	4 (5%)
	Not treated in previous 12 months	1 (1%)

^a^Head and neck, multiple myeloma, and bladder cancer: 2 cases each; colon, liver, cervical, and bladder/prostate cancer: 1 case each.

^b^Multiple responses were possible.

### Perceptions of the Impact of Cancer on QoL

For the aspects of QoL that were most impacted by cancer and its treatment, the theme that was most frequently cited was physical well-being, cited by 52 of 82 (63%) patients. In addition, impact on activities of daily living and emotional well-being were also frequently mentioned, by 25 of 82 (30%) patients each. The most frequent responses cited in the physical-well-being theme were fatigue (26 citations), difficulty getting about (13 citations), and pain (12 citations). The most frequent responses cited in the activities-of-daily-living theme were shopping (10 citations), washing (6 citations), and do-it-yourself/gardening (5 citations). The most frequent responses cited in the emotional-well-being theme were mood (18 citations), stress/anxiety (8 citations), and solitude (4 citations). A full listing of the themes evoked is provided in Table 3.

**Table 3:** Themes of quality of life most impacted by cancer (N=82).

Theme		Number of citations, n	Number of patients citing theme, n (%, 95% CI)
**Physical well-being**			
	Total	74	52 (63%, 53%-74%)
	Fatigue	26	—^a^
	Difficulty getting about	13	—
	Pain	12	—
	Difficulty sleeping	6	—
	Difficulty breathing	4	—
	Difficulty in the morning	4	—
	Concentration	2	—
	Weight gain	2	—
	Incontinence/diarrhea	2	—
	Loss of appetite	1	—
	Sensitivity to changes in the weather	1	—
	Falling ill more often	1	—
**Activities of daily living**			
	Total	36	25 (30%, 21%-41%)
	Shopping	10	—
	Washing/dressing	6	—
	Gardening/jobs in the house	5	—
	Cleaning	4	—
	Driving	4	—
	Cooking	3	—
	Daily activities	3	—
	Keeping appointments	1	—
**Emotional well-being**			
	Total	33	25 (30%, 21%-41%)
	Daily morale	18	—
	Stress/anxiety	8	—
	Loneliness	4	—
	Motivation	1	—
	Fear of dying	1	—
	Feeling helpless	1	—
**Leisure activities**			
	Total	23	21 (26%, 16%-35%)
	Sport/physical activity	11	—
	Going walking	6	—
	Leisure	3	—
	Dancing	2	—
	Traveling	1	—
**Social and family life**			
	Total	21	20 (24%, 15%-34%)
	Outings	6	—
	Family	6	—
	Sex life	4	—
	Seeing friends	3	—
	The way people look at me	1	—
	Conversation	1	—
**Professional life**			
	Total	6	6 (7%, 2%-13%)
**Others**			
	Total	9	9 (11%, 4%-18%)
	Long-term planning	2	—
	Autonomy	2	—
	Wasting time	2	—
	Finding a doctor	1	—
	Not doing anything any more	1	—
	Organization	1	—

^a^Not applicable.

### Discussion of QoL with HCPs

Overall, 75 of 82 (91%) patients considered it important to discuss their HRQoL with an HCP, with 58 (71%) considering it very important and a further 16 (20%) considering it quite important. In addition, 62 of 82 patients (76%) patients had discussed their HRQoL with an HCP at least once. Of the 20 patients who had not done so, 9 (45%) would have liked to, 4 (20%) were not interested, and the remaining 7 (35%) did not know. In addition, 45 of 82 (55%) patients were always or often satisfied with the way in which their HRQoL had been discussed. The principal reasons for satisfaction were that the discussion had resulted in practical solutions being identified (26/45, 58%) and a good relationship with the HCP due to their human qualities (24/45, 53%). Of the 82 patients, 17 (21%) were, however, frequently dissatisfied with this discussion. Reasons for dissatisfaction were insufficient time available for discussing HRQoL (9/17, 53%), a lack of information and explanations provided by the HCP (5/17, 29%), and a lack of empathy on the part of the HCP (4/17, 24%). In addition, 29 of 82 (35%) patients considered that they had been listened to when discussing their HRQoL, whereas an identical number considered that they had not been sufficiently listened to or given the chance to express themselves.

### HCPs Involved in HRQoL Discussions

Patients reported discussing HRQoL with a variety of different HCPs, with the majority reporting multiple points of contact. On average, patients reported consulting 6.7 (95% CI 6.2-7.2) different types of HCPs and discussing HRQoL with, on average, 5.8 (95% CI 5.3-6.3) of these. The most frequently cited HCPs were the general practitioner, the oncologist or radiologist, and the hospital nurse (Table 4). It should be noted that certain HCPs who are frequently consulted, such as community nurses and pharmacists, less frequently discuss HRQoL, whereas other HCPs generally do discuss this issue, even though they are less frequently consulted, such as psychiatrists or palliative care physicians. For 34 of the 53 (64%) patients discussing HRQoL with their oncologist or radiologist, the discussion was initiated by the patient rather than by the physician. When patients were asked with which sort of HCP it was important to discuss HRQoL, the oncologist or radiologist and the general practitioner were the 2 professions that were most often cited, followed by other specialist physicians, the psychiatrist or psychologist, and the hospital nurse (Table 4).

**Table 4:** Health care professionals discussing quality of life with patients (N=82).

Type of HCP^a^	Number of patients who consulted indicated HCP, n (%, 95% CI)	Number of patients who discussed HRQoL^b^ with indicated HCP^c^, n (%, 95% CI)	Number of patients who considered indicated HCP important for discussions of HRQoL, n (%, 95% CI)
General practitioner	57 (70%, 60%-80%)	54 (66%, 56%-76%)	44 (54%, 43%-64%)
Oncologist or radiologist	56 (68%, 58%-78%)	53 (65%, 54%-75%)	54 (66%, 56%-76%)
Community pharmacist	55 (67%, 57%-77%)	42 (51%, 40%-62%)	9 (11%, 4%-18%)
Hospital nurse	54 (66%, 56%-76%)	50 (61%, 50%-72%)	17 (21%, 12%-30%)
Other specialist physician	52 (63%, 53%-74%)	47 (57%, 47%-68%)	21 (26%, 16%-35%)
Community nurse	46 (56%, 45%-67%)	36 (44%, 33%-55%)	10 (12%, 5%-19%)
Surgeon	40 (49%, 38%-60%)	32 (39%, 29%-50%)	15 (18%, 10%-27%)
Psychiatrist or psychologist	31 (38%, 27%-48%)	25 (30%, 21%-41%)	19 (23%, 14%-32%)
Palliative care physician	25 (30%, 21%-40%)	19 (23%, 14%-32%)	13 (16%, 8%-24%)

^a^HCP: health care professional.

^b^HRQoL: health-related quality of life.

^c^Patients stated that they had discussed HRQoL at least once with indicated HCPs.

Of 62 patients, 8 (13%) reported that they had discussed their HRQoL in settings other than medical consultations, such as with patient support groups, discussion groups, or patient groups organized by a nurse.

### Opportunities for Discussing QoL

**Figure 1:**
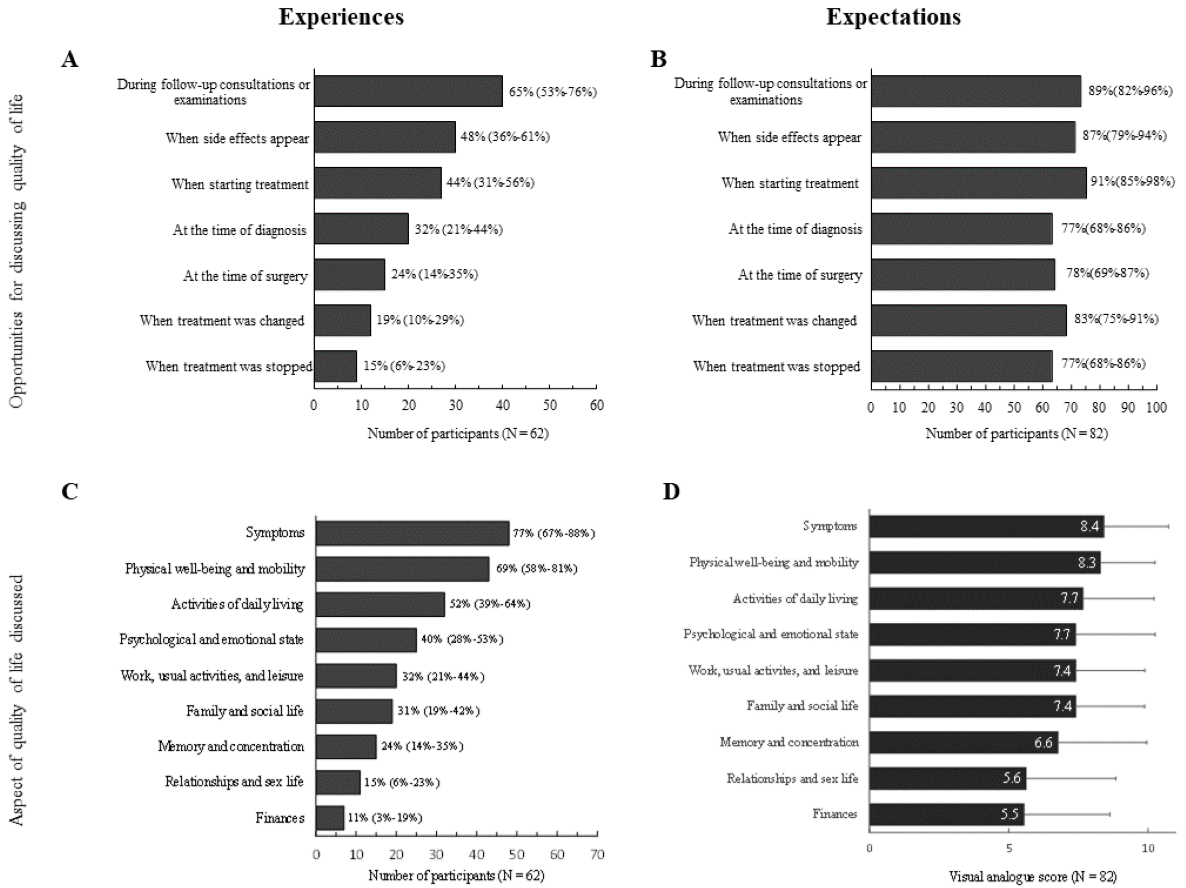
Opportunities for discussing QoL and the aspects of QoL discussed. QoL: quality of life.

QoL was most frequently discussed during follow-up consultations (40/62, 65%) and less frequently at the time the diagnosis was made (27/62, 32%). In particular, HRQoL was addressed when patients reported experiencing side effects or when a new treatment was initiated (Figure 1A). However, most of the patients considered that it was also important to discuss HRQoL at the time of diagnosis (63/82 [77%] expected vs 26/82 [32%] experienced) and to maintain a dialogue throughout their treatment, notably when starting treatment (75/82 [92%] expected vs 36/82 [44%] experienced) and when changes were made to treatment (68/82 [83%] expected vs 16 [19%] experienced) (Figure 1A,B).

### Dimensions of QoL Discussed

Multiple dimensions of HRQoL were usually discussed, with 27 of 62 (47%) patients having discussed 4 or more dimensions. The most frequently discussed dimensions were symptoms, and physical well-being and mobility (Figure 1C). These dimensions were also those that patients thought that it was important to discuss (Figure 1D). However, expectations remained high even for dimensions less frequently addressed, such as memory and concentration, relationships and sex life, or finances.

### Measures for Improving Discussions of QoL

The ways that the health care team could be more attentive to HRQoL that were spontaneously cited most commonly were a better dialogue or a more personal relationship with the HCP (26 citations), more support and guidance (15 citations), and having more personalized information about the disease and treatment (14 citations). With respect to specific measures (Table 5), the most frequently selected were better follow-up of the side effects of treatment (31/82, 38%), the provision of consultations specifically devoted to HRQoL (30/82, 37%), and better coordination of care within the health team (28/82, 34%).

**Table 5:** Specific measures for improving dialogue about the quality of life (N=82).

Theme	Number of patients citing theme, n (%, 95% CI)
Better follow-up of side effects	31 (38%, 27%-48%)
Specific QoL^a^ consultation	30 (37%, 26%-47%)
Better coordination of care	28 (34%, 24%-44%)
Therapeutic education/patient groups	23 (28%, 18%-38%)
Better training on QoL for HCPs^b^	23 (28%, 18%-38%)
Tools for discussing QoL	22 (27%, 17%-36%)
Discussion group/patient support group	14 (17%, 9%-25%)
Longer consultations	13 (16%, 8%-24%)
Systematic involvement of a psychiatrist	12 (15%, 7%-22%)
Involvement of a social worker	10 (12%, 5%-19%)
Other^c^	3 (4%, 0%-8%)

^a^QoL: quality of life.

^b^HCP: health care professional.

^c^One case each of no special needs, patient in survival stage, more resources and time for hospital staff.

## Discussion

### Principal Findings

The results of this study highlight the importance of discussions of HRQoL between patients with cancer treated, or previously treated, with ICIs and their HCPs throughout the treatment journey. Overall, 75 of 82 (91%) patients reported that it was quite or very important to discuss their HRQoL with an HCP. In practice, HRQoL was discussed with an HCP in the majority of cases (62/82, 76%), and most of these patients (45/82, 55%) were satisfied with the quality of the dialogue. Nevertheless, an important gap remains between patients’ expectations and real-life practice, with a significant minority of patients (19/62, 31%) who were either dissatisfied with the way their HRQoL had been discussed or would have liked to have had an opportunity to discuss it. The gap is even more significant in that around half of the patients who had discussed their HRQoL with an HCP (29/62, 35%) felt that they had not been listened to sufficiently or given the chance to express themselves fully.

Many studies have emphasized the beneficial effects that internet use for health issues can have on the doctor-patient relationship, by bringing the “informed patient” to play a more active role in the care process and by facilitating communication [34,35]. Patients using the internet believe that this allows them to understand their disease and its treatment better and, to a lesser extent, helps them take better care of themselves and to participate more in decision making concerning their health [36]. Informed patients also appear to be more motivated to engage in lifestyle changes to maximize the effects of the prescribed treatment [37]. Patients participating in patient forums, such as the Carenity cancer community, are likely to be more proactive in looking for information or support and may have specific expectations for the quality of care that they receive. For this reason, they may have been more likely to initiate discussions of HRQoL than patients who do not participate in such forums. They may also have higher expectations from these discussions and thus be more frequently dissatisfied. However, these assumptions could not be evaluated in this study.

This study was conducted from the patient perspective, and it would be of interest to complement these findings with a similar survey of the importance and utility of discussing HRQoL from the perspective of the HCP. This could help identify areas of convergence between patients and HCPs, as well as understanding the gap between experiences and expectations. For example, an HCP survey could help explain why some areas of HRQoL that are considered important by patients, such as memory problems, relationships, and finances, are rarely addressed by HCPs.

The study revealed that only one-third of patients discussed HRQoL issues related to their work, daily activities, and leisure activities and only 1 in 10 discussed the impact of the cancer on their finances. Since many cancer patients treated with ICIs may achieve durable survival, these treatments may allow a more rapid return to work of cancer patients and a reduction in the amount of sick leave [28], which would be expected to be accompanied by an improvement in HRQoL. The availability of ICI therapy was quite recent at the time of the study. With a longer period of patient follow-up, it would be interesting to evaluate how HRQoL perceptions may evolve over the long term in patients treated with ICIs and in particular to compare perceptions of HRQoL between patients starting ICIs and long-term survivors previously treated with ICIs.

The study findings have identified several important but unfulfilled expectations for a more satisfying dialogue about HRQoL that are widely expressed by patients. Given the importance of monitoring QoL for the management of cancer, integrating a productive dialogue about HRQoL into routine clinical practice is essential, and this study suggests a number of ways in which such a dialogue could be improved so that patients’ expectations are more fully met. Such initiatives are all the more justified in the light of many studies that have reported significant clinical benefits associated with considering the patient’s perceptions of HRQoL [7-13].

First, it would be important to broaden the discussion of HRQoL and not just focus on symptoms or side effects. Only a minority of patients discussed their emotional well-being or the impact of their cancer on their family, social life or professional life, even though they rated highly the importance of discussing these subjects. This focus on symptoms and side effects at the expense of a broader approach to HRQoL has already been emphasized in previous studies of the patient-physician dialogue in patients with advanced cancer receiving standard chemotherapy [38]. Second, the dialogue should be initiated at the time of diagnosis, rather than waiting until a patient has an issue with symptoms or treatment side effects, and continued over the course of the disease. It may be appropriate to set aside specific consultations, or at least a dedicated time during a routine consultation, to talk about HRQoL. Third, the entire care team should be involved in discussion of HRQoL. From the patient’s point of view, the oncologist is the key HCP for discussing HRQoL. However, sharing information about HRQoL across the care team is important to ensure optimal coordination of care. In particular, the general practitioner is also considered an important partner for discussing QoL by patients and could thus play an active role in monitoring HRQoL over the long term.

Physician education should emphasize the need to open the discussion of HRQoL with their patients proactively and systematically. In addition, getting the patient to complete an HRQoL questionnaire before each consultation may be useful for the physician to assess any evolution of HRQoL and to identify any specific issues to be discussed. Different feasibility studies are underway to systematically collect ICI-related symptom and HRQoL data [39,40] and should provide interesting complementary information. A recent study on social media suggested that existing standard HRQoL questionnaires should be enriched with new items that are more relevant for patients treated with ICIs in their daily experience with disease and treatment [29]. Participation in discussion groups or patient support programs could also be systematically proposed. Finally, technological advances now allow monitoring of HRQoL at home through a telemedicine approach using electronic patient-reported outcomes available on applications for smartphones or computers [25,40-42].

### Strengths and Limitations

Like all studies, this one had a number of strengths and limitations. The study included patients with many different types of cancer (principally lung cancer, lymphoma, and melanoma) representative of the principal cancers treated with ICIs in France. The study sample was relatively representative of the target population in terms of age, gender, geographical area, and the type of care received. No data were collected on stage, since there was a doubt as to whether this information could be reliably ascertained from the panel without medical ascertainment, since the patient may not remember and because the stage might have evolved between the time of treatment and the time of the survey. Likewise, panelists were not asked about the specific ICI prescribed, although the list of treatments was specified in the questionnaire, and treatments used by patients in the Carenity platform are not documented in the platform database. For these reasons, it was not possible to investigate the representativeness of responders further, nor to evaluate how these factors might influence perceptions of HRQoL. The number of patients was also relatively small, and patients were unlikely to be representative of all patients with cancer treated or eligible for treatment with ICIs in France. This diversity of cancer types may mask specific HRQoL issues that are important in particular forms of cancer.

Since ICIs were only approved for locally advanced or metastatic cancers at the time of the study, the patient population was at an advanced stage of disease, with one-fifth of patients having been diagnosed for at least 5 years. This implies that all patients should be at a similar stage of their disease, with a current or at least recent experience of ICI therapies. This would ensure relative homogeneity of patients. However, since it may be difficult and arbitrary for patients to distinguish their HRQoL experience with different individual treatments that were managed by the same care providers, patients were invited to describe their experiences over the whole duration of their care since diagnosis. It was thus not possible to interpret patient perceptions and expectations as relating specifically to the period of treatment with ICIs. It was nonetheless possible that recent experiences may dominate earlier ones due to a recall effect.

### Conclusion

In conclusion, this study identified a gap between expectations and reality in the quality of the dialogue between patients and HCPs about HRQoL and also suggested ways to narrow this gap. Patients with cancer have a legitimate desire for a comprehensive and constructive dialogue with their physicians about their QoL, and in the case of patients receiving immunotherapy, this dialogue may be expected to continue for long periods. To meet patient expectations, the dialogue should consider all dimensions of HRQoL. A dialogue about HRQoL should be integrated into clinical practice at every step of the care pathway on a continuous basis from diagnosis to palliative care. It could be facilitated operationally by new modes of care provision, for example, offering specific consultations with an HCP dedicated to discussing HRQoL. Optimizing this dialogue should thus be a priority for physicians treating patients with cancer.

## Abbreviations

EORTC-QLQ: European Organisation of Research and Treatment of Cancer Quality of Life Questionnaire
FACT: Functional Assessment of Cancer Therapy
HCP: health care professional
HRQoL: health-related quality of life
ICI: immune checkpoint inhibitor
QoL: quality of life
